# Effect of the Lapping Platen Groove Density on the Characteristics of Microabrasive-Based Lapping

**DOI:** 10.3390/mi11080775

**Published:** 2020-08-14

**Authors:** Taekyung Lee, Haedo Jeong, Sangjik Lee, Doyeon Kim, Hyoungjae Kim

**Affiliations:** 1 Dongnam Regional Division, Korea Institute of Industrial Technology, Busan 46938, Korea; blayer@kitech.re.kr (T.L.); sjiklee@kitech.re.kr (S.L.); kdykim@kitech.re.kr (D.K.); 2 School of Mechanical Engineering, Pusan National University, Busan 46241, Korea; hdjeong@pusan.ac.kr

**Keywords:** groove density, boundary lubrication, lapping characteristic, oil film, sapphire substrate, microabrasive

## Abstract

Microabrasive-based lapping is widely used in the manufacturing of single-crystal substrates such as sapphire, SiC, and GaN. Although many studies have been conducted to improve the lapping process characteristics, most of them focused on process conditions or consumables. In this study, the effect of the lapping platen groove density on the lapping characteristics was studied using a sapphire substrate. Groove density was defined as the ratio of groove width to groove pitch, and the displacement of the lapping head was measured to calculate the oil film thickness. It was confirmed that, for groove densities below 0.30, hydroplaning occurs when the oil film thickness increases. When the oil film thickness is larger than the abrasive particle size, the material removal rate is low because the abrasive does not participate in the lapping process. When the oil film was developed, the experimental results showed a high surface roughness and poor flatness of the substrate, as only large abrasive particles participated in the lapping process. Therefore, to improve the lapping characteristics, it is important to reduce the groove density by reducing the groove pitch, which prevents the development of the oil film.

## 1. Introduction

**Figure 1:**
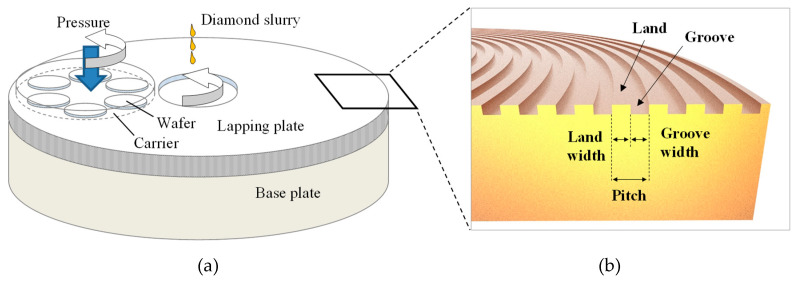
Schematics of (**a**) the lapping process and (**b**) groove definition.

Lapping using microabrasives improves the flatness of the substrate being machined and reduces the thickness of the subsurface damaged layer in the manufacturing of single-crystal substrates such as sapphire, SiC, and GaN, which in turn reduces the post processing time of chemical mechanical polishing (CMP) [1,2,3,4]. Microdiamonds are normally used as the abrasive particles, and metal platens made of cast iron, copper, or tin are used as the lapping platen [5,6,7,8]. In the lapping process, as shown in Figure 1a, the substrate is machined using the relative motion between the substrate and the platen. A pressure load is applied to the substrate while supplying a uniformly dispersed abrasive solution (i.e., a slurry) on the platen surface. The flatness of the platen is adjusted and the grooves on the platen surface are formed using a facing device [9,10].

The lapping characteristics are represented by the material removal rate (MRR), the substrate surface roughness, and the flatness of the substrate. The MRR is an indicator of lapping efficiency and generally represents the decrease in thickness of the substrate per unit time [11]. The surface roughness is a factor related to the subsurface damaged layer of the substrate. In general, the lower the surface roughness, the smaller the thickness of the subsurface damaged layer. The flatness of the substrate is represented by the total thickness variation (TTV), which is the difference between the maximum and minimum thicknesses in the measurement area [12,13,14]. These lapping characteristics are complexly influenced by various factors, such as the rotational speed of the platen, the pressing force, the slurry properties, the lapping platen shape, the platen grooves density, etc. [15,16,17,18]. Yuan [19] and Lee [20] studied the relationship between the material removal mechanism of the abrasive and the MRR. Chen et al. [21] and Tanaka et al. [22] determined the effects of the mechanical properties of the lapping platen on the MRR and surface roughness. Shi [23] and Tseng [24], among other researchers, studied the effect of process conditions, such as pressure and velocity, on the MRR. Moreover, many studies have been conducted to improve the lapping process characteristics [25,26,27,28]. However, most of these studies focused on process conditions; therefore, there is not sufficient knowledge about the effect of the platen grooves on the aforementioned characteristics.

The platen grooves are depressions created on the platen surface, as shown in Figure 1b. The section of the platen surface that is in contact with the substrate is called “the land,” and the groove spacing is represented by the groove pitch. In the lapping process, spiral or concentric grooves are mainly used, and their shape is rectangular to maintain the same contact area between the platen and the substrate, even when the abrasion of the grooves progresses. The grooves remove debris generated during the process and increase the surface area of the platen to facilitate the control of the platen temperature. The groove is also closely related to the lapping characteristics because it greatly affects the dispersion of particles and the flow of the slurry at the contact interface between the platen and the substrate [29]. Studies on the change of the hydrodynamic pressure and contact pressure with the pad groove shape in the CMP process [30] and on the change in the oil film thickness with the CMP process conditions [31] have been conducted. However, the effect of the grooves on the contact interface between the substrate and the platen in the microabrasive-based lapping process has not been studied yet.

To address this knowledge gap, in this study, the effect of groove density on the state of the contact surface between the substrate and the platen, i.e., the magnitude of the oil film thickness and frictional force, were investigated and the effect of the change in the oil film thickness on lapping characteristics, including MRR, surface roughness, and substrate flatness, was studied.

## 2. Materials and Methods

### 2.1. Definition of Groove Density

Concentric square grooves were selected to study the effect of groove density on the oil film thickness and the lapping process characteristics. To vary the groove density, the groove width (*G*) was fixed at 2.0 mm and the groove density (*D*_g_) was modified by adjusting the groove pitch (*P*) and land width (*L*). Groove density is defined as:

(1) $$D_{g}=\frac{\mathrm{Groove}\;\mathrm{width}}{\mathrm{Pitch}}=\frac{G}{P}=\frac{P\;-\;L}{P}$$

The depth of the grooves was designed to be 1.0 mm, and the effect of groove density on the oil film thickness and the lapping characteristics was studied through experiments in which the groove density was varied from 0.10 to 0.69. The groove design parameters used are presented in Table 1.

### 2.2. Experimental Equipment and Conditions

**Figure 2:**
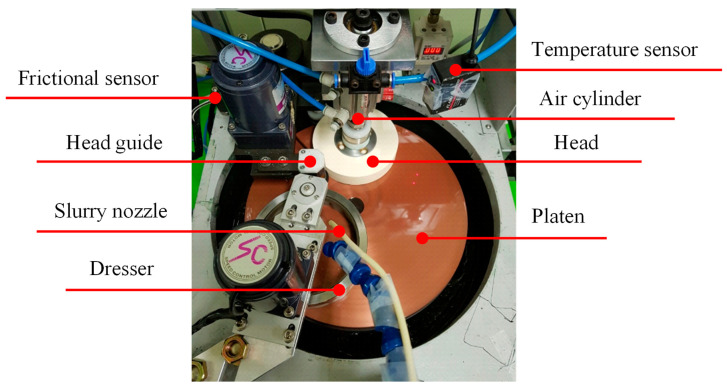
Experimental equipment for microabrasive-based lapping.

Test equipment was developed for conducting experiments to identify the lapping characteristics according to the groove density, as shown in Figure 2. This equipment consists of a head for fixing the wafer, a head guide for rotating the head, and an air cylinder for pressing the substrate. The slurry nozzle supplies the slurry onto the platen, and the dresser distributes the slurry evenly over the platen. A sapphire substrate with a diameter of 100 mm was used as the hard substrate, and polycrystalline diamonds with an average particle size of 3.1 μm were used as the abrasive particles. A resin–copper platen made by sintering copper powder and resin was used as the lapping platen. The experimental conditions used, including the pressure applied to the substrate, the rotational speed of the platen, and the flow rate of the slurry, were fixed to consider only the groove density change effect. The experimental conditions adopted are detailed in Table 2. The platen surface was finished via a facing process before lapping the substrate. After facing, a stabilization process was performed for 10 min under the same conditions used in the lapping experiments to exclude the effects of platen surface roughness and platen flatness on the results.

To measure the substrate thickness, a contact displacement sensor (GT2-A12KL, Keyence, Osaka, Japan) having a resolution of 0.1 μm was used. The average thickness was obtained by measuring the substrate thickness of 21 points, and the MRR was calculated by dividing the processing time by the change in average thickness. The surface roughness of the substrate was measured using a noncontact surface roughness measuring device (NewView 7300, ZYGO, Middlefield, CT, USA), and the surface roughness value was calculated by averaging the surface roughness values of 21 points after lapping. The platen surface was analyzed using a scanning electron microscope (S-4800, Hitachi, Tokyo, Japan).

**Figure 3:**
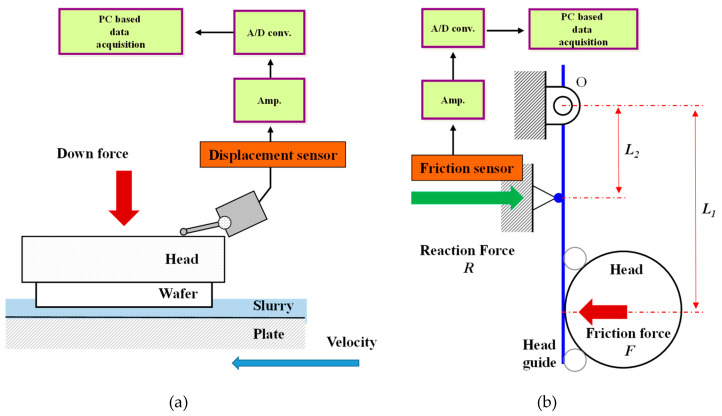
Schematics of measuring method: (**a**) the oil film thickness and (**b**) the frictional force.

To determine the condition of the contact interface between the substrate and the platen during the lapping process, an oil film thickness measuring system was used; its configuration is shown in Figure 3a. The sensor used to measure the film thickness was an electric induction type displacement sensor (1318 inductive indicator, Mahr, Goettingen, Germany) with a resolution of 0.1 μm. The state in which no slurry was supplied on the platen surface was defined as the dry state, and the state in which the slurry was supplied on the platen surface was defined as the wet state. In the dry state, the displacement of the rotating head in contact with the rotating platen was measured for 10 s. Next, by measuring the displacement of the lapping head for 30 s under the same conditions used in the wet-state lapping experiment, the average displacement during the last 10 s was calculated. Finally, the average head displacement difference between the dry and wet states was calculated to obtain the oil film thickness.

To confirm the oil film thickness changes, the frictional force between the lapping platen and the substrate was measured using a frictional force measuring system, as shown in Figure 3b. Piezoelectric force sensor (9132B, Kistler, Winterthur, Switzerland) was used to measure the frictional force. The frictional force acting on the lapping head, to which the substrate was bonded, was transmitted to the head guide, amplified using a hinge, and measured by the friction sensor. The frictional force was calibrated using a push–pull gauge before performing the experiment.

## 3. Experimental Results and Discussion

### 3.1. Change of Oil Film Thickness According to Groove Density

**Figure 4:**
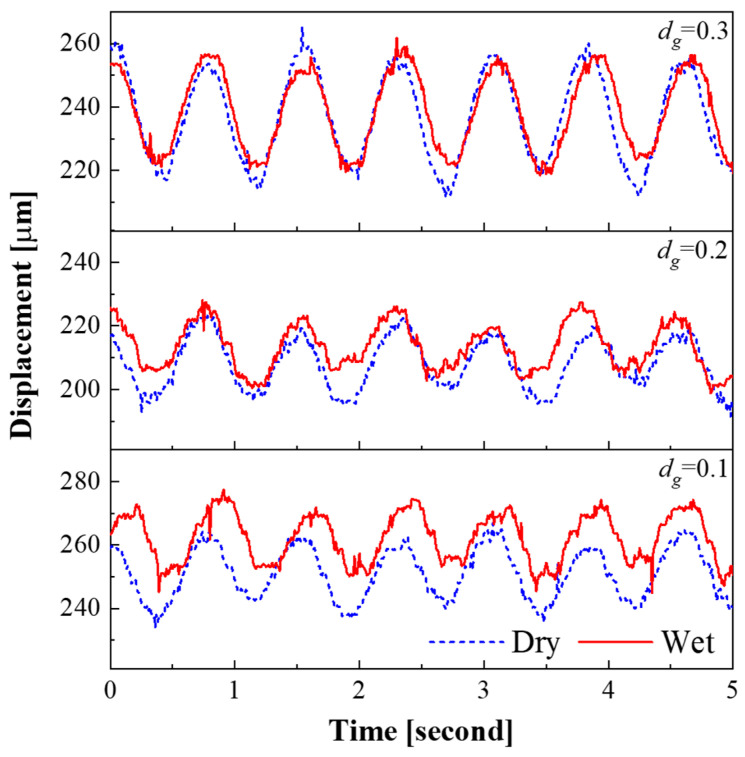
Displacement of the head with contact conditions and the groove density.

**Figure 5:**
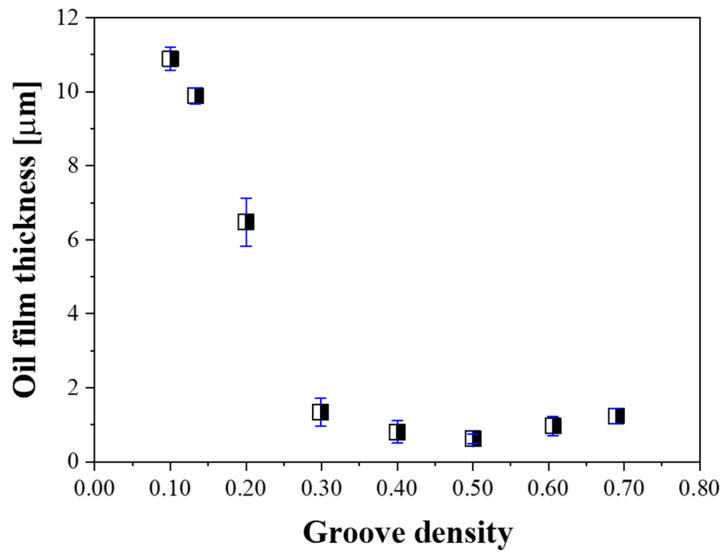
Effect of groove density on oil film thickness.

The oil film thickness between the platen and the substrate was measured to confirm the contact interface state change with groove density during lapping process. Figure 4 shows the effect of the groove density on the displacement of the polishing head. When the groove density was 0.10, it showed the largest displacement difference, and as the groove density increased, the displacement difference decreased. In the case when groove density was 0.30, there was almost no displacement difference. When the groove density was 0.10, the average displacements of the head in the dry and wet states were 251 and 262 µm, respectively. The average displacement of the head increased by 11 µm when slurry was supplied on the platen, indicating that an 11 µm thick oil film was formed. In this way, the oil film thickness for all groove density values was measured, as shown in Figure 5.

The oil film thickness was highest when the groove density was 0.10 and gradually decreased as the groove density increased. When the groove density was 0.30 or higher, the oil film thickness converged to approximately 1 µm. This indicates that when the groove density was less than 0.30, hydroplaning occurred at the contact interface between the substrate and the platen, resulting in the development of the oil film.

### 3.2. Change of Frictional Force According to Groove Density

**Figure 6:**
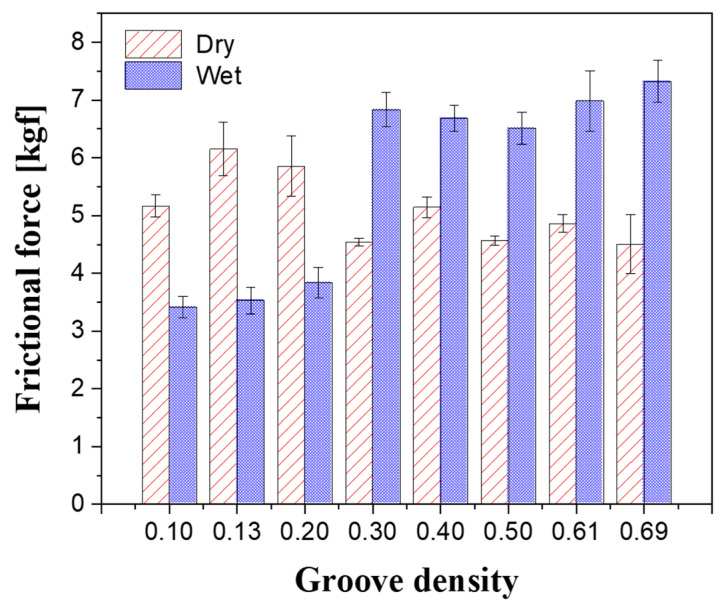
Effect of groove density on frictional force.

To verify the formation of the oil film, the frictional force generated between the substrate and the platen was measured at each groove density value under the same experimental conditions used for oil film thickness determination; the average values of the measured frictional forces are shown in Figure 6. In the dry state, the friction force decreased with increasing the groove density and tended to converge at a groove density of 0.3 or above. In the wet state, the frictional force increased up to a groove density of 0.3 and showed a convergent tendency. The significant frictional force differences between the dry and wet states indicate that the contact interface condition changed. When the groove density ranged from 0.10 to 0.20, the frictional force in the dry state was greater than that in wet state, i.e., when slurry was supplied between the substrate and the platen, the frictional force decreased. As indicated by the oil film thickness results presented previously, when the groove density was low, the oil film developed and the substrate and the platen were completely separated by the slurry. Conversely, when the groove density ranged from 0.30 to 0.69, the frictional force in the wet state was greater than that in the dry state. This is because the diamond abrasives on the land generated cutting forces while removing the sapphire substrate.

**Figure 7:**
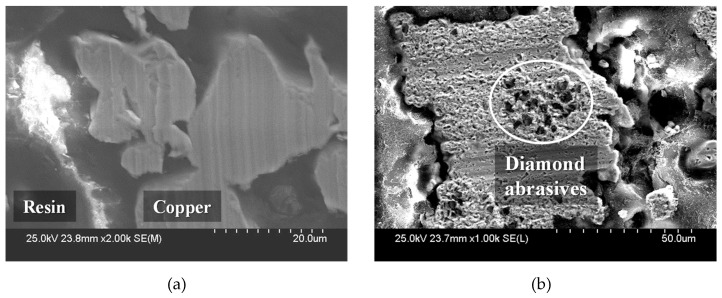
Scanning electron microscope (SEM) images of the lapping platen’s land: (**a**) after the facing process and (**b**) after the lapping process.

By comparing the changes in oil film thickness and frictional force, when the groove density was less than 0.3, it can be assumed that the contact interface experienced mixed lubrication, in which the pressure load was supported by the abrasive particles and the slurry, and the smaller the groove density, the larger the oil film thickness. When the groove density was 0.3 or higher, boundary lubrication occurred, in which the abrasive particles on the platen surface were in contact with the substrate. In the boundary lubrication region, the load applied to the substrate is supported by the abrasive particles. In addition, some large particles are deeply embedded in the platen or broken when subjected to a force higher than the breaking strength due to the characteristics of the polycrystalline diamond particles [32]. The scanning electron microscope (SEM) image of the land presented in Figure 7a shows that only tool marks are present on the copper surface after the facing process. As shown in Figure 7b, after the lapping process, indentation marks are generated when the abrasive particles are rolled between the substrate and the land or when they are momentarily stuck on the land. In addition, some particles are embedded on the copper surface.

**Figure 8:**
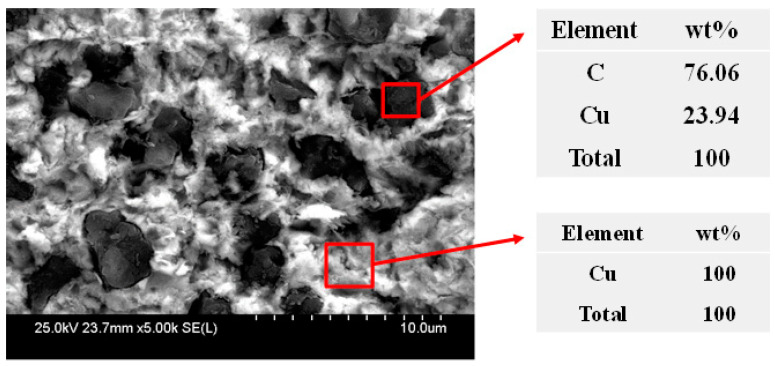
Energy dispersive analysis of X-ray (EDAX) results after lapping.

Energy dispersive analysis of X-ray (EDAX) was performed to confirm what the particles on the surface were. The results are shown in Figure 8. It was confirmed that the particles are mostly composed of carbon and are diamond abrasives. Owing to the diamond abrasive particles embedded on the land, the measured oil film thickness was approximately 1 µm, even when the groove density was 0.3 or higher. From these results, we can conclude that, depending on the groove density, a change in the oil film thickness at the contact interface may occur, which may affect the lapping process characteristics.

### 3.3. Effect of Oil Film Thickness on Lapping Characteristics

**Figure 9:**
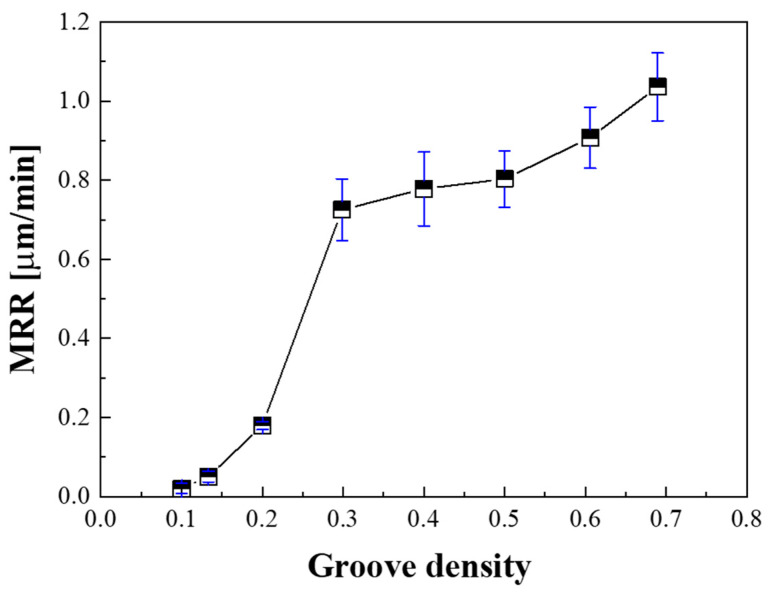
Material removal rate (MRR) as a function of groove density.

To confirm the effect of the oil film thickness change on the lapping process characteristics, lapping experiments were performed for each groove density. The MRR was calculated by dividing the processing time by the amount of removed material; the results obtained are shown in Figure 9. The MRR presented its lowest value at a groove density of 0.1, increased rapidly at a groove density of 0.3, and gradually increased at groove densities above 0.3. This is because when the groove density is 0.3 or less, the load pressing the substrate is mostly supported by the slurry, in a mixed lubrication state. In addition, since the average size of the diamond abrasive particles used for lapping (3.1 µm) was lower than the oil film thickness, they could not participate in the lapping process. Consequently, as the groove density decreased, the MRR decreased rapidly.

**Figure 10:**
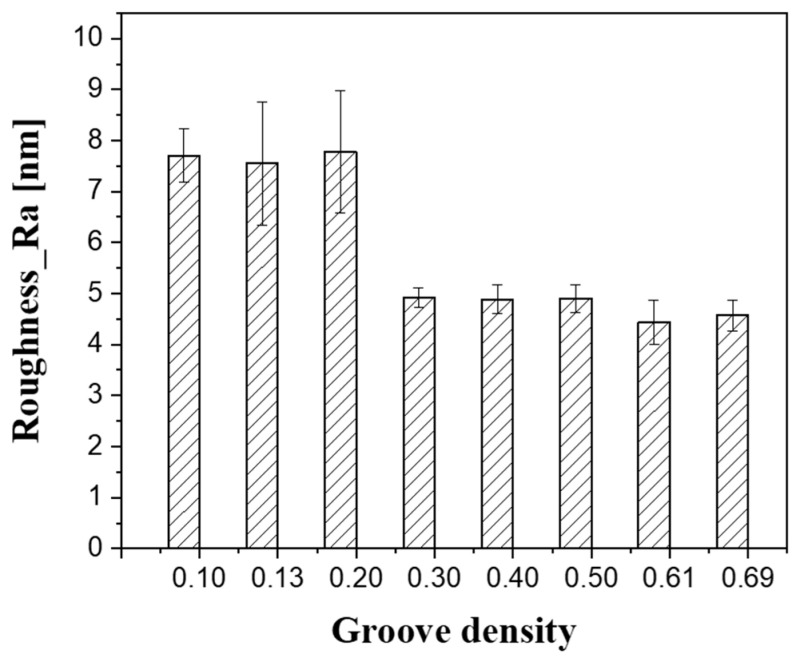
Substrate surface roughness as a function of groove density.

Figure 10 presents the average value of the surface roughness measured at 21 points after lapping at each groove density. When the groove density was smaller than 0.30, at which the oil film was thick, the surface roughness (Ra) was approximately 7.5 nm; when the groove density was 0.30 or higher, the surface roughness rapidly decreased to less than 5.0 nm. The particles in the diamond abrasive had an average size of 3.1 μm; however, their size was normally distributed, ranging from 1 to 5 μm. In the case of low groove densities (0.10–0.20), most of the abrasive particles could not participate in the lapping process because the oil film thickness between the substrate and the platen was greater than the abrasive particle size. As only large abrasive particles could be in contact with the substrate, some of them created deep and wide scratches on the substrate surface. In addition, when the oil film thickness changed owing to the runout of the platen, some particles came in contact with the substrate under a high load, resulting in a low MRR and a high surface roughness.

**Figure 11:**
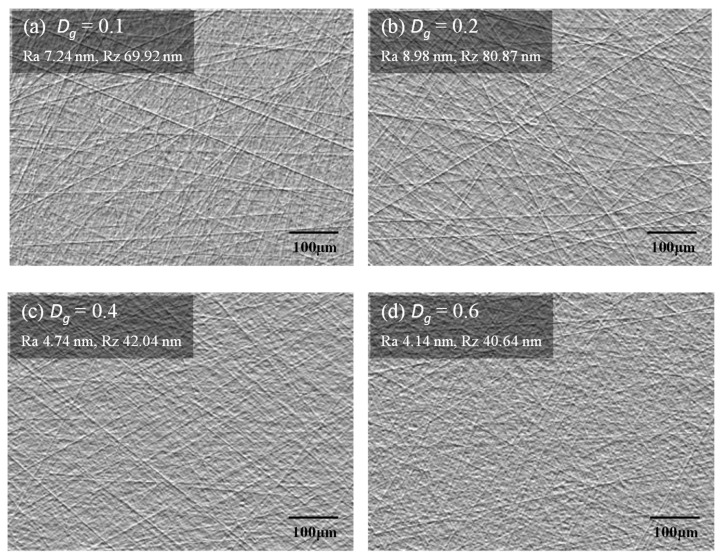
Differential interference contrast images of scratches on the substrate surface.

This can be confirmed through the differential interference contrast images of the substrate surface presented in Figure 11; in these images, scratches are shaded and darkened as their depths increase. In Figure 11a,b, as the groove density was low, many deep and wide scratches are distributed on the substrate surface. For groove densities greater than 0.30, shallow, narrow, and fine scratches are densely distributed on the substrate surface, as shown in Figure 11c,d.

**Figure 12:**
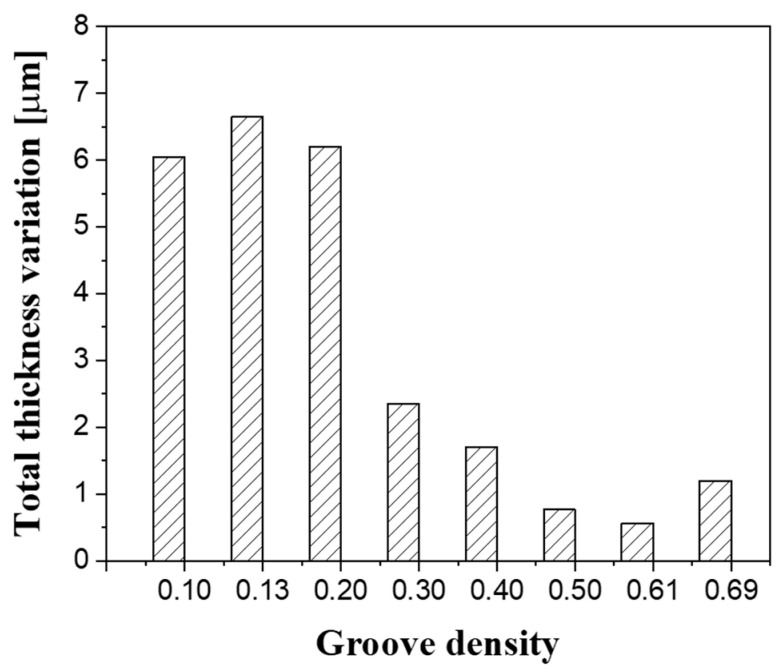
Effect of groove density on the total thickness variation.

**Figure 13:**
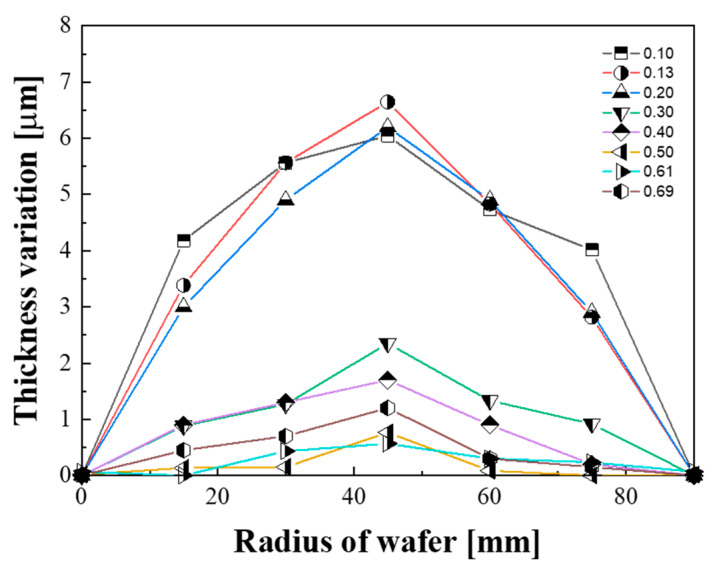
Effect of groove density on the wafer shape.

The change in the film thickness caused by groove density variation also affected the flatness (i.e., the TTV) of the substrate after lapping. As shown in Figure 12, when the groove density was less than 0.30, the TTV of the substrate was approximately 6 μm; conversely, when the groove density was greater than 0.30, it presented small values (from 1 to 2 μm). In the lapping process, the flatness of the substrate is mainly determined by the shape of the platen [33,34,35]. In this study, the shape of the platen was machined equally at all groove densities via a facing process. However, as shown in Figure 13, the flatness of the substrate presented different characteristics depending on the groove density. When the groove density was less than 0.30, the shape of the substrate was convex, with the largest thickness deviation located at the center of the substrate. The edge effect that the edge thickness of the substrate becomes thinner is a very important factor because it is related to the yield in the semiconductor manufacturing process [36]. When the groove density is less than 0.30, the edge effect occurs and the flatness of the substrate is deteriorated.

**Figure 14:**
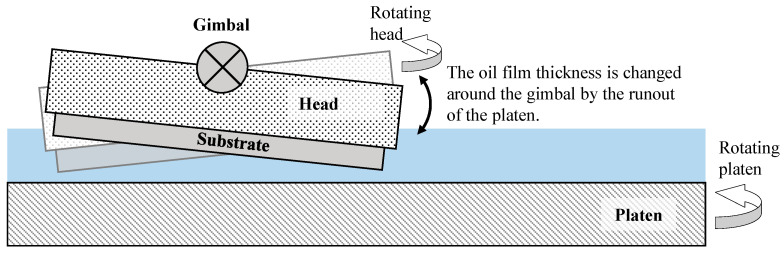
Schematic of oil film thickness change owing to platen runout.

Thakurta [37] presented the contact interface between the substrate and the pad as a solid-fluid-solid contact in the CMP process. This study confirms that since the head has a gimbal structure, the moment due to frictional force is unstable, which can lead to hydrodynamic lubrication and mixed lubrication at the same time. This phenomenon may occur because the lapping head used in this experiment also has a gimbal structure. The removal amount at the edge of the substrate increased as the oil film thickness changed around the head gimbal owing to the runout of the platen, as shown in Figure 14. If the groove density is greater than 0.30, this phenomenon does not occur because the platen and the substrate are attached to each other in the boundary lubrication state.

## 4. Conclusions

To determine the effect of groove density on the lapping process characteristics, the change in the oil film thickness at the contact interface between the substrate and the platen was investigated. When the groove density was less than 0.3, a mixed lubrication state was observed; moreover, it was confirmed that the smaller the groove density, the thicker the oil film formed. When the groove density was 0.3 or higher, a boundary lubrication state was observed because the platen, the abrasive particles, and the substrate were in direct contact. In the friction force measurements, even when the groove density was less than 0.3, the frictional force in the wet state was lower than that in the dry state, since hydroplaning phenomenon occurs between the substrate and the platen. At groove densities above 0.3, it was confirmed that the frictional force in the wet state increased and became larger than that in the dry state owing to the lapping force of the abrasive particles.

The lapping platen groove density affects the lapping process characteristics by changing the oil film thickness at the contact interface between the substrate and the platen. The MRR gradually decreases as the groove density decreases, and then rapidly decreases at groove densities below 0.3, since the load pressing the substrate is mostly supported by the oil film, in a mixed lubrication state. On the contrary, it was confirmed that the surface roughness increases rapidly at groove densities below 0.3. This is because large abrasive particles participate in the lapping process to produce thick and deep scratches when the oil film thickness is large. In addition, since most of the pressure is supported by the oil film, a runout phenomenon in which the lapping head shakes around the gimbal occurs, thereby deteriorating the flatness of the substrate.

Therefore, in order to improve the lapping process characteristics, it is important to increase the groove density by reducing the groove pitch to prevent the development of the oil film. Since the oil film thickness is related to the slurry properties, it is possible to prevent the development of the oil film by adjusting the slurry viscosity. When the groove density is high, it is possible to achieve a boundary lubrication state; however, the contact area of the platen may be reduced, thereby reducing the lifespan of the platen. Therefore, it is important to select an appropriate groove density to achieve the desired lapping characteristics. In further work, it is necessary to study the effects of kinematics factors such as the rotational speed and the pressure load on the oil film thickness, which will enhance the understanding of the hydrodynamic phenomenon occurring at the contact interface between the platen and the substrate.

## Figures and Tables

**Table 1:** Values of the groove parameters used in the experiments.

Parameter	Value							
Groove width (mm)	2							
Land width (mm)	18.0	13.0	8.0	4.7	3.0	2.0	1.3	0.9
Pitch (mm)	20.0	15.0	10.0	6.7	5.0	4.0	3.3	2.9
Groove density	0.10	0.13	0.20	0.30	0.40	0.50	0.61	0.69

**Table 2:** Experimental conditions used in the lapping tests.

Parameter	Condition
Lapping plate	Copper–resin plate, Ø300 mm
Wafer	Sapphire, Ø100 mm
Abrasive	Polycrystalline diamond, 3.1 μm
Slurry flow rate	4 mL/min
Rotational velocity	Head: 80 rpm, plate: 80 rpm
Pressure	39.2 kPa
